# Elucidating the CodY regulon in *Staphylococcus aureus* USA300 substrains TCH1516 and LAC

**DOI:** 10.1128/msystems.00279-23

**Published:** 2023-06-13

**Authors:** Ye Gao, Saugat Poudel, Yara Seif, Zeyang Shen, Bernhard O. Palsson

**Affiliations:** 1 Department of Biological Sciences, University of California San Diego, La Jolla, California, USA; 2 Department of Bioengineering, University of California San Diego, La Jolla, California, USA; 3 Department of Pediatrics, University of California San Diego, La Jolla, California, USA; 4 Bioinformatics and Systems Biology Program, University of California San Diego, La Jolla, California, USA; 5 Novo Nordisk Foundation Center for Biosustainability, Kongens Lyngby, Denmark; Colorado State University, Fort Collins, Colorado, USA

**Keywords:** *Staphylococcus aureus*, CodY, Transcription factor binding sites, Metabolism

## Abstract

CodY is a conserved broad-acting transcription factor that regulates the expression of genes related to amino acid metabolism and virulence in Gram-positive bacteria. Here, we performed the first *in vivo* determination of CodY target genes using a novel CodY monoclonal antibody in methicillin-resistant *Staphylococcus aureus* (MRSA) USA300. Our results showed (i) the same 135 CodY promoter binding sites regulating the 165 target genes identified in two closely related virulent *S. aureus* USA300 TCH1516 and LAC strains; (ii) the differential binding intensity for the same target genes under the same conditions was due to sequence differences in the same CodY-binding site in the two strains; (iii) a CodY regulon comprising 72 target genes that are differentially regulated relative to a CodY deletion strain, representing genes that are mainly involved in amino acid transport and metabolism, inorganic ion transport and metabolism, transcription and translation, and virulence, all based on transcriptomic data; and (iv) CodY systematically regulated central metabolic flux to generate branched-chain amino acids (BCAAs) by mapping the CodY regulon onto a genome-scale metabolic model of *S. aureus*. Our study performed the first system-level analysis of CodY in two closely related USA300 TCH1516 and LAC strains, revealing new insights into the similarities and differences of CodY regulatory roles between the closely related strains.

**IMPORTANCE:**

With the increasing availability of whole-genome sequences for many strains within the same pathogenic species, a comparative analysis of key regulators is needed to understand how the different strains uniquely coordinate metabolism and expression of virulence. To successfully infect the human host, *Staphylococcus aureus* USA300 relies on the transcription factor CodY to reorganize metabolism and express virulence factors. While CodY is a known key transcription factor, its target genes are not characterized on a genome-wide basis. We performed a comparative analysis to describe the transcriptional regulation of CodY between two dominant USA300 strains. This study motivates the characterization of common pathogenic strains and an evaluation of the possibility of developing specialized treatments for major strains circulating in the population.

## INTRODUCTION

*Staphylococcus aureus* is a ubiquitous, Gram-positive pathogen that causes a diverse range of bacterial infections from skin and soft-tissue infections to potentially fatal ones, such as pneumonia, endocarditis, osteomyelitis, sepsis, and toxic shock syndrome (1). Coupled with the growing prevalence of methicillin-resistant *S. aureus* (MRSA) strains, the worldwide threat posed by this pathogen remains critical (2, 3). Current research provides insights into important features of these strains, including antibiotic resistance and extensive virulence factors. So far, a number of known virulence factors consist of surface-associated proteins (4). To combat the worldwide spread of *S. aureus*, significant effort is being focused on the investigation of the transcription factors that control virulence factors during infection (5, 6). With different strains circulating in the population and the serial rise and fall of specific strain types, it is important to understand the fundamental differences between them (7).

CodY is an important broad-acting transcription factor in *S. aureus* (8, 9). While the primary known role of CodY is to regulate the metabolic genes in response to the changes in cellular branched-chain amino acid and GTP concentrations, it also controls the expression of several virulence factors acting as a bridge between metabolism and virulence (8, 10 - 13). The CodY regulon, estimated to consist of 150 to more than 200 genes, can vary in size between *S. aureus* strains, and CodY can even have opposite effects on the expression of the same gene in different strains (10, 12). At present, the direct determination of CodY DNA-binding sites using ChIP-exo and the identification of the corresponding target genes have not been achieved.

To address this challenge, we developed a reliable ChIP-grade monoclonal antibody and conducted *in vivo* genome-wide experiments (ChIP-exo) to identify the 165 identical CodY target genes in two common *S. aureus* USA300 isolates (TCH1516 and LAC). To reconstruct the CodY regulon, we compared RNA-seq profiles of the wild-type strain and *codY* mutant. To examine the network-level effects of CodY, we used a genome-scale metabolic model to simulate the flux state of central carbon metabolism, demonstrating the regulatory activities of CodY to generate branched-chain amino acids (BCAAs). Our study used a comprehensive pipeline to characterize the CodY regulon between closely related USA300 substrains that included genetic parameters and network-level computational models.

## RESULTS

### Comparative genomic analysis of *S. aureus* USA300 reveals a high level of identity between two closely related strains, TCH1516 and LAC

The community-associated methicillin-resistant *S. aureus* (CA-MRSA) clones belonging to the USA300 lineage have become the dominant sources of MRSA in the United States (14). They are distinct from other *S. aureus* clones such as USA200 (UAMS-1), USA400 (MW2), and ST8 MSSA (Newman) (14). Two dominant CA-MRSA USA300 strains, TCH1516 and LAC, isolated in Houston and Los Angeles, respectively, were chosen to study CA-MRSA clones as they represent well-characterized strains in the USA300 lineage (15). These strains have become the epidemic clones spreading in the community (16).

**Fig 1:**
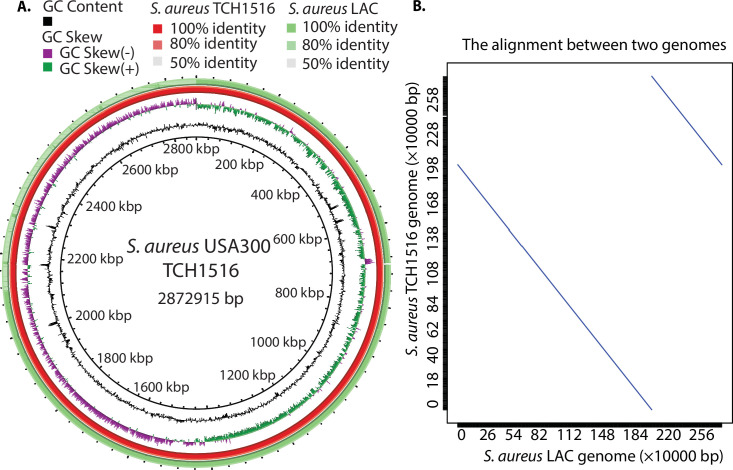
The comparison of two dominant *Staphylococcus aureus* USA300 isolates (TCH1516 and LAC). (**A**) Circular representation of whole-genome comparison of *S. aureus* TCH1516 (red ring, reference strain) and LAC strains (green ring). Each ring of the circle represents a specific complete genome that corresponds to different colors in the legend above. The similarity between strains is represented by the intensity of the color. Darker colors represent higher similarities than lighter ones. Deleted regions are represented by blank spaces inside the circles. The whole-genome comparisons were generated by BRIG. Alignment identity cutoffs of 0.8 (upper) and 0.5 (lower) were used to determine missing regions in the query genome (*S. aureus* LAC) compared to the *S. aureus* TCH1516 reference. Since *S. aureus* TCH1516 was the first genome to be annotated in the USA300 lineage, it was used as a reference genome for this study. (**B**) Dot plot of a nucleotide-based alignment of the genomes between *S. aureus* USA300 TCH1516 and LAC.

To understand the similarity between two strains, using whole-genome alignment, we characterized the genomes of TCH1516 and LAC, having lengths of ~2.872 Mb and ~2.878 Mb, respectively (Table 1). According to NCBI Prokaryotic Genome Annotation Pipeline (PGAP) (17), we found a high level of identity at the nucleotide level between these two closely related strains, and on average, they displayed 99.5% identity for all coding genes (Fig. 1A). Furthermore, MUMmer, a system for rapidly aligning large DNA sequences to one another, was used to check for the synteny between the strains (18). We produced a MUMmer dot plot resulting from the alignment of their chromosomal sequences, demonstrating that they have high similarity between the genomes and large-scale inversions located at positions ~2.18 Mb (TCH1516) and ~2.20 Mb (LAC) (Fig. 1B). Overall, these data revealed that they have a high degree of sequence similarity throughout their genome sequences.

**TABLE 1:** Comparison of annotation features in *S. aureus* USA300 lineage

	*S. aureus* TCH1516	*S. aureus* LAC
Total sequence length (bp)	2,872,915	2,878,171
Total chromosome and plasmids	3, NC_010079.1 (chromosome) NC_012417.1 (plasmid 1) NC_010063.1 (plasmid 2)	2, CP035369.1 (chromosome) CP035370.1 (plasmid)
Gene (total)	2920	2892
CDS (total)	2841	2802
Genes/CDS (coding)	2763	2733
RNA		
rRNAs (5S, 16S, 23S)	6, 5, 5	7, 6, 6
tRNAs	59	67
ncRNAs	4	4
Pseudo gene (total)	78	69

### Genome-wide identification of CodY-binding sites in *S. aureus* USA300 TCH1516

Previously, 59 enriched CodY-binding regions that corresponded to 49 transcriptional units in the *S. aureus* NCTC 8325 were identified *in vitro* deploying an affinity purification experiment using purified *S. aureus* His-tagged CodY and a related mutant strain (10). However, there are no direct *in vivo* measurements of the interaction between CodY and DNA in the recent USA300 isolates. Thus, using a novel monoclonal antibody, we performed chromatin immunoprecipitation followed by exonuclease digestion (ChIP-exo) to identify the CodY-binding sites with single-nucleotide resolution in *S. aureus* USA300 TCH1516 under RPMI with 10% LB medium, as it is more closely mimicking the nutritional environment of the human host (19).

**Fig 2:**
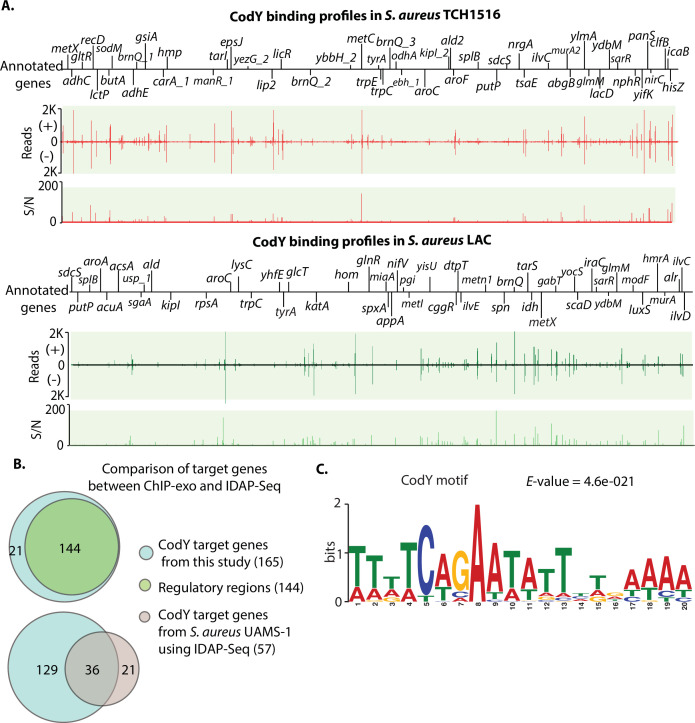
Comparison of CodY-binding sites between TCH1516 and LAC strains. (**A**) Identification of CodY DNA-binding sites in *S. aureus* substr. USA300 TCH1516 and LAC at the genome. S/*N* denotes signal-to-noise ratio. (+) and (−) indicate forward and reverse reads, respectively. Upper panel: an overview of CodY-binding profiles across the *S. aureus* TCH1516 genome at mid-exponential growth phase in RPMI 1640 + 10% LB medium. Bottom panel: an overview of CodY-binding profiles across the *S. aureus* LAC strain genome at mid-exponential growth phase under RPMI 1640 + 10% LB medium. (**B**) Distribution of *in vivo* CodY genome-wide binding sites at the genome of the TCH1516 strain (upper panel). Comparison of CodY-binding sites obtained from this study (ChIP-exo) with CodY target genes from *S. aureus* USA200 UAMS-1 using IDAP-Seq (bottom panel). (**C**) The consensus DNA sequence for *S. aureus* TCH1516 CodY-binding motif.

Using a peak calling algorithm (MACE), a total of 165 CodY target genes were identified from TCH1516 (Fig. 2A, full data set in Table S3). Of the 49 transcriptional units previously identified as strong candidates for direct regulation by CodY in *S. aureus* UAMS-1 using IDAP-Seq, 73% (36/49) were also detected in TCH1516. Compared to the IDAP-Seq approach, this study detected the interaction between TF-DNA *in vivo* to enrich the DNA bound by CodY in its natural state but also showed the location of each peak at single-nucleotide resolution.

Our results showed that 88% (144/165) of CodY-binding sites were located within the intergenic regions, and the remaining 12% of binding sites were found in coding regions (Fig. 2B, upper panel). Most of the binding sites located in intergenic regions were present upstream of assigned genes, indicating that CodY may play critical regulatory roles in the expression of these genes. A total of 129 novel CodY target genes were identified (Fig. 2B, bottom panel). These findings expanded the list of CodY target genes in TCH1516 and enabled a better understanding of the global regulatory role of CodY in the USA300 lineage.

### Identification of the CodY-binding motif in *S. aureus* TCH1516

To identify the DNA sequence motif of CodY-binding sites, we used the MEME motif-searching algorithm with the genomic sequences of binding sites, and then we identified the conserved 20 bp CodY-binding motif, which was consistent with the previously characterized CodY DNA-binding consensus sequence (AATTTTCWGAAAATT) in *S. aureus* UAMS-1 (Fig. 2C). Furthermore, the *S. aureus* TCH1516 CodY-binding motif is similar with the CodY-binding motif (AATTTTCWGAATATTCWGAAAATT) reported in *Listeria monocytogenes* and *Bacillus subtilis* (20, 21). These results suggest that CodY likely has a conserved DNA-binding domain in Gram-positive bacteria (Fig. S1).

### Comparison of *in vivo* CodY-binding sites in *S. aureus* TCH1516 and LAC strains

As two community-associated methicillin-resistant strains, *S. aureus* TCH1516 and LAC have an identical CodY sequence (Fig. S2). To investigate the direct gene targets of CodY, we employed the same monoclonal antibody to perform the ChIP-exo assay in *S. aureus* LAC under RPMI with 10% LB medium (19).

*S. aureus* LAC has 165 CodY target genes identified at the genome that were identical to those from the TCH1516 strain, though their positions at each chromosome are different due to the inversions mentioned earlier (Fig. 2A, bottom panel, Table S3). The alignment of the binding motifs revealed that the two strains have 18 nucleotides that overlap (*P*-value = 9.35e-09) by using TOMTOM. Among the 165 target genes, there are 10 genes directly related to virulence in *S. aureus* TCH1516 and LAC strains (Table 2), consistent with the report that CodY links metabolism with virulence gene expression (8). Taken together, these data demonstrated that the global regulator CodY controls the expression of metabolism and virulence genes in *S. aureus* (11).

**TABLE 2:** The CodY target genes considered to be virulence factors in the TCH1516 and LAC strains

Virulence factors	Gene	*S. aureus* TCH1516	*S. aureus* LAC
Cell wall-associated fibronectin-binding protein	*ebh*	Yes	Yes
Clumping factor B	*clfB*	Yes	Yes
Extracellular adherence protein/MHC analogous protein	*eap/map*	Yes	Yes
Intercellular adhesin	*icaB*	Yes	Yes
Hyaluronate lyase	*hysA*	Yes	Yes
Lipase	*geh*	Yes	Yes
Serine protease	*splB*	Yes	Yes
Sbi	*sbi*	Yes	Yes
Type VII secretion system	*esaG*	Yes	Yes
Enterotoxin-like K	*selk*	Yes	Yes

### Strain-specific binding intensity is associated with DNA sequence variations in the binding site of CodY

As chromatin immunoprecipitation with high-throughput sequencing is widely used to identify genome-wide binding sites, many studies have initiated the investigation of the variation in the binding intensities of the same transcription factors between different conditions or homologous transcription factors across different strains. Though the genome-wide CodY-binding sites identified were the same in the two strains, we found that strain-specific binding peaks had differing binding intensities (Fig. S3), which provided the case study to explore the causes of the variations of the binding intensities between two closely related strains.

To fully evaluate whether the strain-specific binding peaks are due to the changes in the sequence-specific affinity to which CodY is bound, we first identified a 20-bp motif based on a merged set of CodY-binding peaks from the strains using MEME (22) (Fig. S4). We then utilized the computational method MAGGIE to measure differences in the DNA sequence in paired binding sites in the two strains (23). MAGGIE used two-sided Wilcoxon signed-rank tests to associate epigenomic changes at homologous sequences with motif mutations to identify motifs that likely contribute to the strain-specific difference. By performing the analysis on a consensus motif of both strains, we could eliminate the computation bias toward any one type of strain.

**Fig 3:**
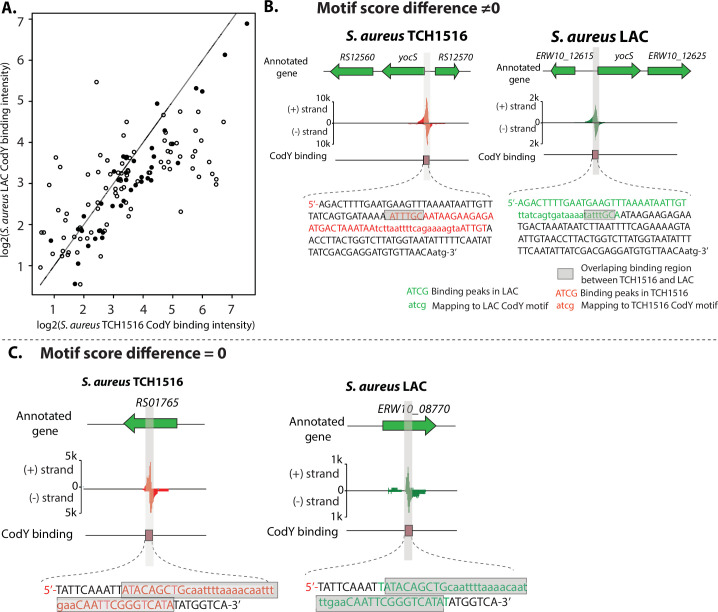
Differential CodY-binding peaks area plot for TCH1516 and LAC highlighting binding sites with identical binding motifs. (**A**) Binding peak areas of log2(TCH1516_binding_intensity) (x-axis) and log2(LAC_binding_intensity) (y-axis) are shown. The diagonal line represents identical peak areas. The 45 binding sites with near zero sequence difference (Fig. S5) are shown with the solid dots, while those that are significantly different are shown with open circles. (**B**) Case study I: the binding peak at the upstream of gene *yocS*, which has non-zero motif score difference between TCH1516 and LAC. Annotations of color code nucleotides are shown in the legend. Nucleotides in red and green represent the CodY peak sequences in TCH1516 and LAC, respectively. Gray denotes the overlap between a pair of peak sequences. (**C**) Case study II: the binding peak at the gene USA300HOU_RS01765, which has zero motif score difference between TCH1516 and LAC.

Among 135 pairs of peaks between the strains, 45 of 135 (33.3%) binding sites with identical DNA-binding sequences had a range of motif score difference between −1 and 1 (Fig. S5). The pairs of peak heights for the 135 shared binding sites in LAC and TCH1516 were plotted (Fig. 3A). When we highlighted the 45 binding sites with near identical DNA sequences, we observed that they represent the dots closest to the 45-degree line. This indicates that these 45 sites have similar peak heights in the two strains. The binding sites where the DNA sequence differs are off the 45-degree line, showing that these differences lead to differential peak heights for the same binding site in the two strains.

Furthermore, to visualize the differences between a pair of binding peaks, we mapped CodY-binding peaks to the reference genome. For example, the sequence of the peak located at the upstream of the gene *yocS* in TCH1516 slightly (7 bp) overlaps the corresponding peak sequence in LAC (Fig. 3B). Another example is from a pair of peaks from unknown genes USA300HOU_RS01765 and ERW10_08770. They had zero motif score difference, and thus we observed that both peak sequences nearly overlap each other (Fig. 3C).

### Genome-wide reconstruction of CodY regulons in the *S. aureus* USA300 lineage

The ChIP-exo data sets from this study expanded the number of target genes in the *S. aureus* USA300 lineage to 165, which included 129 novel CodY target genes. Of these, 37% (47 of 129) were metabolic genes, which were non-essential genes in *S. aureus* USA300 (24).

**Fig 4:**
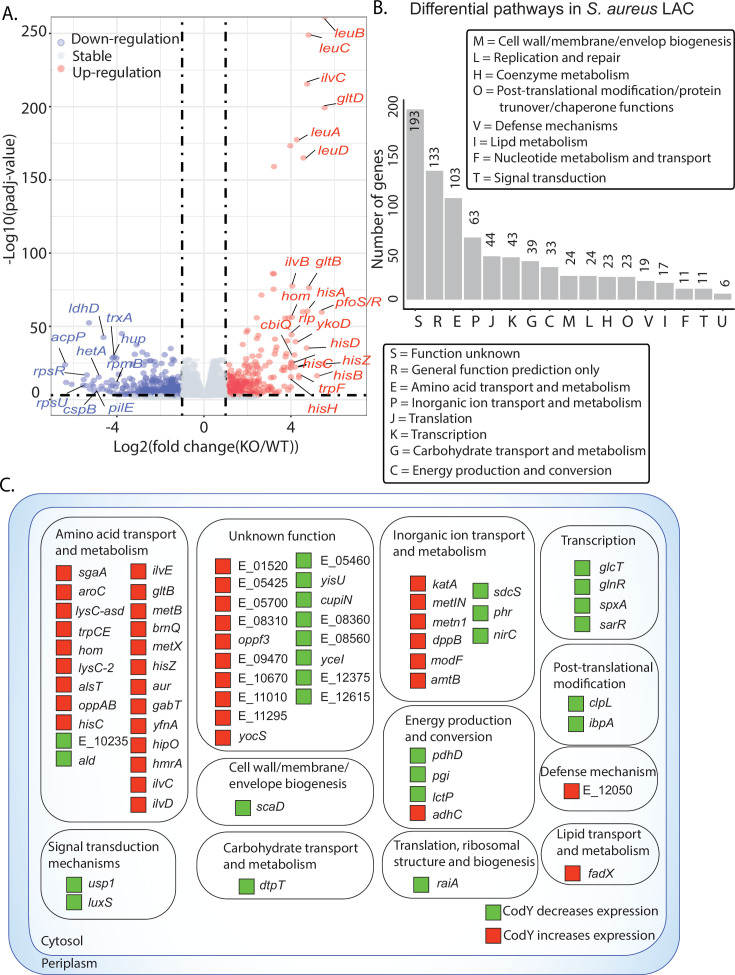
Role of CodY regulon in *S. aureus* USA300. (**A**) A volcano plot representation of differential expression analysis performed between wild type and *codY* mutant. (**B**) Functional enrichment analysis by Clusters of Orthologous Groups (COGs) classification of 809 differentially expressed genes in the *S. aureus codY* mutant compared to wild type. The number of genes are based on the annotated genome. The top six enriched pathways were amino acid transport and metabolism, inorganic ion transport and metabolism, translation, ribosomal structure and biogenesis, transcription, carbohydrate transport and metabolism, and energy production and conversion. The functional enrichment was analyzed by performing the hypergeometric test. The asterisk indicates hypergeometric *P*-value < 0.05. (**C**) Reconstruction of 72 CodY regulons in the *S. aureus* USA300.

To further characterize the regulatory roles of CodY in the *S. aureus* USA300 lineage, we compared gene expression profiling of the *codY* mutant to that of the wild type under RPMI with 10% LB medium, and we found that there were 809 genes differentially expressed in the *codY* mutant [at least twofold change (*P* < 0.05) in RNA-seq expression] (Fig. 4A). The majority of genes related to metabolism were up-regulated in the *codY* mutant, which was consistent with the repressor role that CodY plays in *S. aureus* (25). Furthermore, we used functional enrichment analysis by Clusters of Orthologous Groups (COGs) classification of 809 differentially expressed genes (DEGs). Of 809 DEGs, 193 are of the unknown function category; of those with known function, we found the top six differential enrichment pathways: amino acid transport and metabolism, inorganic ion transport and metabolism, translation, ribosomal structure and biogenesis, transcription, carbohydrate transport and metabolism, and energy production and conversion (Fig. 4B).

These 809 DEGs comprise the CodY regulon—a set of genes directly or indirectly regulated by CodY in the USA300 LAC strain. Prior studies integrated a global transcription factor DNA binding with expression profiles of cells lacking that same TF compared to wild-type cells to reconstruct the regulons of the transcription factor (26, 27). In this study, we integrated CodY-binding sites with transcriptomics to reconstruct the direct regulons of CodY—those genes that showed a change in expression when CodY was deleted and also had a corresponding binding peak upstream. Accordingly, we identified 72 direct regulons of CodY in the LAC strain (Fig. 4C) (28). Over half of the direct regulons (51%, 37 of 72) were related to the metabolic pathways (amino acid transport and metabolism, inorganic ion transport and metabolism, and energy production/conversion). In addition, 57% (41 of 72) of regulons were negatively regulated by CodY in the wild type. The remaining DEGs (737 of 809) were indirect regulons—those genes that show a change in expression when CodY is deleted but do not have a corresponding binding peak upstream.

Recent studies introduced an independent component analysis (ICA)-based framework in the bacteria that decomposes a compendium of RNA-sequencing (RNA-seq) expression profiles to determine the underlying regulatory structure and defined the term iModulon to describe the condition-invariant sets of genes regulated by the transcription factor. Each iModulon is calculated based on an ICA of RNA-seq data, which allows for a top-down determination of gene expression patterns (29). CodY iModulons were calculated based on RNA-seq data from *S. aureus* USA300 strains in the prior study (30). Importantly, we compared CodY direct regulons from the experimental data with the reported CodY iModulon from ICA-based machine learning and found that 90% (65/72) of direct CodY regulons significantly overlap with CodY iModulons, which confirmed the regulatory roles of CodY (Fig. S6). The CodY iModulon contains a set of genes having significant overlap with previously predicted CodY regulons.

Furthermore, CodY regulons were directly involved in signal transduction mechanisms, transcription, translation, post-translational modification, and defense mechanisms. These data indicated that CodY contributes to global regulatory roles beyond the metabolism of *S. aureus* USA300.

### Rerouting of flux through central carbon metabolism to generate the BCAAs

CodY is reported to regulate the expression of metabolic genes in response to changes in the pools of specific metabolites, i.e., the branched-chain amino acids (BCAAs); isoleucine, leucine, and valine (ILV); and nucleoside triphosphate (GTP), to regulate genes involved in the biosynthesis of these amino acids (31). Despite responding primarily to ILV levels, CodY also regulates the expression of genes involved in biosynthesis of other amino acids, such as threonine, histidine, and aspartate. Therefore, we sought to understand how *S. aureus* manages flux through these CodY regulated biosynthetic enzymes during ILV starvation.

**Fig 5:**
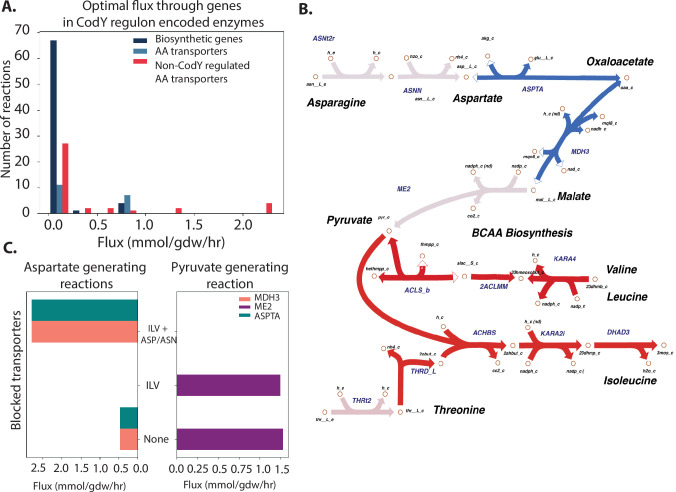
Central metabolic flux rerouting to generate branched-chain amino acids. (**A**) Optimal flux through CodY-regulated biosynthetic enzymes, transporters, and non-CodY-regulated transporters in RPMI medium. Y-axis represents the number of reactions with a given flux. A histogram showing fluxes through different categories of reactions (i.e., CodY-regulated biosynthetic enzymes, CodY-regulated AA transporters, and non-CodY-regulated transporters). When amino acids are present, *S. aureus* imports them via transporters (light blue and red bars). (**B**) pFBA solution of *S. aureus* grown in RPMI without ILV predicts rerouting of several central carbon metabolic fluxes to generate ILV precursors. Red arrows represent reactions with increased flux during ILV starvation, and blue arrows represent those with decreased flux relative to ILV rich conditions. Malate dehydrogenase and aspartate synthase (blue arrows) lower flux when BCAA transporters are blocked. Note: Full ILV biosynthesis pathways are not shown. (**C**) A histogram showing the number of reactions under different nutrient conditions. X-axis shows different conditions (different amino acids conditions, i.e., none, ILV, ILV + ASP/ASN). Double y-axis: left y-axis shows the flux; right y-axis shows the number of reactions. Flux through pyruvate generating malate enzyme (ME2) and aspartate generating malate dehydrogenase (MDH3) and aspartate transaminase (ASPTA) are dependent on the presence of aspartate and asparagine in the media.

In order to understand the partition of fluxes required for ILV synthesis, we performed parsimonious Flux Balance Analysis (pFBA) using the metabolic model iYS854 of *S. aureus* USA300 (32, 33). pFBA determines metabolic fluxes that maximize growth while minimizing the sum of fluxes through the system (32). As expected, simulated growth in RPMI supplemented with ILV led to growth with zero flux through the CodY regulated biosynthetic pathway in favor of direct transport of the necessary metabolites (Fig. 5A). Restricting flux through all ILV transporters (see Materials and Methods) led to a spike in flux through the ILV biosynthetic enzymes. Interestingly, in ILV starvation conditions, aspartate, a precursor to isoleucine biosynthesis, was not generated by the TCA cycle intermediate oxaloacetate. Instead, aspartate was derived from the breakdown of asparagine, while flux through the TCA cycle was redirected toward generating pyruvate (another precursor for ILV biosynthesis) via malate (Fig. 5B). This result indicated that in a BCAA-deficient environment with sufficient asparagine and aspartate, *S. aureus* generates pyruvate to increase precursor pools available for isoleucine biosynthesis. Indeed, flux through the two reactions linking the TCA cycle and aspartate, malate dehydrogenase (MDH3), and aspartate transaminase (ASPT) increased when aspartate and asparagine transporters were blocked in addition to BCAA transporters (Fig. 5C). Flux through malic enzyme (ME2) was only detectable in the presence of aspartate and asparagine, i.e., when the flux through those enzyme transporters were not blocked. Taken together, our pFBA analysis demonstrates how fluxes through CodY-regulated biosynthetic enzymes are rerouted in ILV starvation conditions to generate required precursors. It suggests that the unified regulation of genes encoding enzymes involved in various amino acid biosynthesis pathways by CodY is due to the need for *S. aureus* to coordinate the fluxes through these pathways to efficiently generate ILV.

## DISCUSSION

With the increasing number of whole-genome sequences becoming available for multiple strains of a pathogenic species, the importance of the differences in their genomes and gene content is becoming more appreciated (34). Although many properties of pathogenic strains can now be predicted from sequence alone (35 - 37), detailed experimental characterization of differences in multiple strains is also needed. In this study, we combined genome-wide experiments and computational modeling to address the differences of CodY in the dominant CA-MRSA USA300 clinical isolates TCH1516 and LAC (15). The study resulted in a series of significant findings.

First, through the genome-wide identification of *in vivo* binding sites at single-nucleotide level, we found the same 165 CodY target genes in two clinical USA300 strains. Although the prior study by Majerczyk et al. (10) identified 59 regions bound by CodY in *S. aureus* NCTC8325 through *in vitro* DNA affinity technology, those results could not represent the scope of CodY target genes in USA300 strains due to genome sequence differences and pathogenicity between USA300 strains and NCTC8325. In addition, the ChIP-exo method reveals a snapshot of the binding sites obtained in a particular strain under tested conditions. As a global regulator, CodY participated in the regulation of many cellular processes, including amino acid biosynthesis, transport of nutrient molecules, and virulence. Some regulations may require the cofactors to activate the binding affinity between CodY and DNA. Thus, ChIP-exo is appropriate to provide a snapshot of target genes that reflects the *in vivo* status of CodY under the conditions used. Furthermore, it provides a near base pair (bp)-level resolution of protein-DNA interaction, which greatly improves the accuracy of identifying CodY-binding sites at the genome and the sequences of the CodY motif.

Second, an examination of the differential binding intensity of CodY in the promoter regions of the same target genes in the two different strains under the same conditions revealed that the variance was due to DNA sequence differences in the same CodY-binding site in the two strains, while the CodY protein was identical in both strains. This novel finding reveals insights into the system-level analysis of CodY target genes and differential binding intensity across closely related strains.

Third, the study identified 10 virulence genes that belong to different types of virulence factors, which demonstrated that CodY connects metabolism genes with virulence genes in *S. aureus* (8). In addition, our combined ChIP-exo and RNA-sequencing analysis also shed light on how CodY affects *agr* expression. As previously described and confirmed by our ChIP-exo data, CodY represses the expression of *agr* locus but does not bind strongly to the *agr* promoter (13). However, our data also show that CodY both binds to the *sarR* promoter and the expression level of sarR gene goes down in codY knock-out mutant. The product of this gene, SarR, is a repressor of the *agr* locus, which suggests a possible avenue by which CodY is able to repress *agr* expression (38). It is worth noting that the LAC strain used for gene expression profiling in this study expresses *agr* at high levels even in the exponential phase, and therefore it was not significantly affected by codY knockout as previously noted. Considering that different *S. aureus* lineages may have distinct virulence factors, we could expand this study to identify many other virulence factor genes coordinated by CodY across different *S. aureus* strains.

Finally, an existing genome-scale model of the metabolic network in *S. aureus* can be constrained by the regulatory action of CodY (33). This model computes the consequent systematic rerouting fluxes through metabolism of BCAAs as a result of CodY regulatory action.

Taken together, this study demonstrates, for the first time, the similarities in the function of a conserved globally acting transcription factor (i.e., CodY) between closely related pathogenic strains of *S. aureus* USA300. Understanding the CodY regulon will allow deciphering the network of CodY controlling the amino acid metabolism and virulence and motivate an evaluation of the possibility of developing appropriate treatments for major strains circulating in the population.

## MATERIALS AND METHODS

### Bacterial strains and culture conditions

The bacterial strains used in this study were described in Table S1. Methicillin-resistant *Staphylococcus aureus* (MRSA) strain substr. USA300 TCH1516 (also named USA300-HOU-MR) was originally isolated from an outbreak in Houston, Texas, and caused severe invasive disease in adolescents (39). MRSA USA300 LAC was originally isolated from the Los Angeles county jail (15). MRSA USA300 LAC contains three small plasmids, one encoding resistance to tetracycline and another encoding erythromycin resistance. The third plasmid is cryptic. For ease of genetic manipulation and to avoid interference, all three plasmids were cured, yielding strain *S. aureus* USA300 JE2. Thus, *S. aureus* JE2, as a parental strain, was used for all sequence-defined Tn mutagenesis experiments. The *codY* mutant was from *S. aureus* JE2 in which each of non-essential genes was disrupted via mariner *bursa aurealis* Tn mutagenesis. *S. aureus* JE2 and a transposon mutant strain that integrated into codY are from Nebraska Transposon Mutant Library. All *S. aureus* strains were grown in tryptic soy broth (TSB, Sigma-Aldrich, St. Louis, MO) or RPMI-1640 (Gibco, Houston, TX) with 10% lysogeny broth (LB, Sigma-Aldrich, St. Louis, MO) containing 10 g/L peptone, 5 g/L yeast extract, and 10 g/L NaCl with shaking (250 rpm) at 37℃, maintaining a flask-to-medium volume ratio of 9:1, unless otherwise specified.

### Protein expression and monoclonal antibody production

The monoclonal antibody used was developed with GenScript (Piscataway, NJ). Briefly, the *S. aureus* USA300 *codY* gene (see Table S2) with 6X His tag was synthesized and cloned into a pET-30a(+) plasmid (EMD Millipore, Burlington, MA) using the Ndel and HindIII sites. The protein was expressed in *E. coli* BL21(DE3) in the Terrific Broth medium containing 50 µg/mL kanamycin. The culture was incubated at 37°C with shaking at 200 rpm. When the optical density at 600 nm (OD_600_) reached about 0.5, the cell culture was transferred to 15°C. Then, protein expression was induced with addition of 0.4 mM IPTG (final concentration) and shaking for an additional 12 h. Then, cells were harvested by centrifugation and lysed by sonication at 4°C, using a tip probe at cycles of 5 s ON and 25 s OFF at a power level at 6.0 until full lysis (Misonix Sonicator 3000 Ultrasonic Cell Disruptor, Cole-Parmer, Vernon Hills, IL). The His-tagged CodY protein was purified as previously described using Ni-NTA affinity columns (Qiagen, Germany) (40). The concentration was determined by the BCA protein assay (Thermo Fisher Scientific, Waltham, MA) with bovine serum albumin as the standard. Using purified His-tagged CodY, monoclonal anti-CodY antibody was generated by injection of CodY into the BALB/C mouse. The custom-made monoclonal antibody (IgG) that binds the CodY protein was produced and purified by GenScript (Piscataway, NJ).

### ChIP-exo experiments

ChIP-exo experimentation was performed following the procedures described previously using a custom-made antibody that binds the CodY protein (27). To identify CodY-binding maps for each strain *in vivo*, the culture with an OD_600_ of ~0.5 was used to generate ChIP-DNA. Formaldehyde was added at a final concentration of 1% and incubated at room temperature for 20 min in a platform rocker. Cross-linking was quenched by adding 250 mM of glycine and incubating for 5 min. Cells were washed three times with ice-cold 1 X PBS (Thermo Fisher Scientific, Waltham, MA). The resulting cells were lysed with Ready-lyse lysozyme solution (Epicentre, Waltham, MA). Lysate was sheared by sonication (Misonix Sonicator 3000) with the settings (the output power level 9.0 for 30 min at 4 degree) to generate 300–500 bp randomly sheared chromosomal DNA fragments. Then, immunoprecipitation was carried out at 4°C with overnight incubation by using a custom-made antibody that binds the CodY protein (GenScript, Piscataway, NJ). In contrast, mouse IgG (MilliporeSigma™ Upstate) was used as internal control. The CodY protein, together with its cross-linked DNA and covalently bound mouse antibody, was captured with 50-µL Dynabeads Pan mouse IgG (Invitrogen) and washed with buffer I [50 mM Tris-HCl (pH 7.5), 140 mM NaCl, 1 mM EDTA, and 1% Triton X-100]. ChIP materials (chromatin beads) were used to perform on-bead enzymatic reactions of the ChIP-exo method (41). The sheared DNA of chromatin beads was repaired by the NEBNext End Repair Module (New England Biolabs) followed by the addition of a single dA overhang and ligation of a first adaptor (5′-phosphorylated) using the dA-Tailing Module (New England Biolabs, Ipswich, MA) and the NEBNext Quick Ligation Module (New England Biolabs), respectively. The first adaptor was designed to have different indices to distinguish different DNA samples after the sequencing. After ligation, multiple ChIP materials could be pooled together. Nick repair was performed by using PreCR Repair Mix (New England Biolabs). Lambda exonuclease- and RecJ_f_ exonuclease-treated chromatin was eluted from the beads and incubated overnight at 65°C to reverse the protein-DNA cross-link. RNAs- and proteins-removed DNA samples were used to perform primer extension and second adaptor ligation with following modifications. The DNA samples incubated for primer extension as described previously (27) were treated with dA-Tailing Module (New England Biolabs) and NEBNext Quick Ligation Module (New England Biolabs) for second adaptor ligation. The DNA sample purified by GeneRead Size Selection Kit (Qiagen) was enriched by polymerase chain reaction (PCR) using Phusion High-Fidelity DNA Polymerase (New England Biolabs). The amplified DNA samples were purified again by GeneRead Size Selection Kit (Qiagen, Germany) and quantified using Qubit dsDNA HS Assay Kit (Life Technologies, Carlsbad, CA). Quality of the DNA sample was checked by running Agilent High Sensitivity DNA Kit using Agilent 2100 Bioanalyzer (Agilent, Santa Clara, CA) before sequenced using HiSeq 2500 (Illumina) following the manufacturer’s instructions.

### Peak calling for ChIP-exo data set

Peak calling was performed as previously described (27). Sequence reads generated from ChIP-exo were mapped onto the reference genome using bowtie (42) with default options to generate SAM output files. MACE program was used to define peak candidates from biological duplicates for each experimental condition with sequence depth normalization (43). To reduce false-positive peaks, peaks with signal-to-noise (S/*N*) ratio less than 1.5 were removed. The noise level was set to the top 5% of signals at genomic positions because the top 5% makes a background level in a plateau and top 5% intensities from each ChIP-exo replicates across conditions correlate well with the total number of reads (27, 44, 45). The calculation of S/*N* ratio resembles the way to calculate ChIP-chip peak intensity where the IP signal was divided by Mock signal. Genome-scale data were visualized using MetaScope (https://sites.google.com/view/systemskimlab/software?authuser=0).

### Motif search from ChIP-exo peaks

The sequence motif analysis for CodY-binding sites was performed using the MEME software suite (22). For each strain, sequences in binding regions were extracted from the reference genome (*S. aureus* TCH1516: GenBank: NC_010079.1, NC_012417.1, and NC_010063.1; *S. aureus* LAC: GenBank: CP035369.1 and CP035370.1). To achieve a more accurate motif, the sequence of each binding site was extended by 10 bp at each end. The width parameter was fixed at 20 bp, and the minsites parameter was fixed at 90% of the total number of the sequence. All other parameters followed the default setting.

### COG enrichment

CodY regulons were categorized according to their annotated COG database (46). Functional groups in core, accessory, and unique CodY-regulated genes were determined by COG categories.

### Multiple genome comparison and alignment

MUMmer was used to run the complete nucleotide-based alignments to check for synteny among the sequences (18). BLAST Ring Image Generator (BRIG) was used to show a genome-wide visualization of coding sequences identity between *S. aureus* USA300 TCH1516 and LAC (47). Multiple genomes were analyzed by the M-GCAT, which is a tool for rapidly visualizing and aligning the most highly conserved regions in multiple prokaryote genomes. M-GCAT is based on a highly efficient approach to anchor-based multiple genome comparison using a compressed suffix graph (48).

### Determining the orthologs between different *S. aureus* strains with bidirectional BLAST hits

To determine the orthologs between different strains of *S. aureus*, core genome-containing conserved genes between the TCH1516 and LAC were first established using bidirectional BLAST hits (49). In this analysis, all protein sequences of CDS from both genomes are BLASTed against each other twice with each genome acting as reference once. Two genes were considered conserved (and therefore part of the core genome) if (i) the two genes have the highest alignment percent to each other than to any other genes in the genome and (ii) the coverage is at least 80%.

### RNA extraction and library preparation

The culture of *S. aureus* JE2 and its derivatives of *codY* mutant were incubated in RPMI+10% LB medium at 37 degree overnight with agitation, and then they were used to inoculate the fresh media (1/200 dilution). The volume of the fresh media was 50 mL for each biological replicate. The fresh culture was incubated in RPMI+10% LB medium at 37 degree with agitation to the mid-log phase (OD_600_ ≈ 0.5). RNA-seq was performed using two biological replicates. Once the cell culture reached the mid-exponential phase (OD_600_ = 0.5), the cell culture was collected for RNA extraction. Transcripts were stabilized by mixing 3 mL of cell cultures at the mid-log phase with 6 mL of RNAprotect Bacteria Reagent (Qiagen). Samples were immediately vortexed for 5 s, incubated for 5 min at room temperature, and then centrifuged at 5000 g for 10 min. The supernatant was decanted and any residual supernatant was removed by inverting the tube once onto a paper towel. Total RNA samples were then isolated using the RNeasy Plus Mini kit (Qiagen) following the manufacturer’s instruction. Samples were then quantified using a NanoDrop 1000 spectrophotometer (Thermo Scientific), and quality of the isolated RNA was checked by running RNA 6000 Pico Kit using Agilent 2100 Bioanalyzer (Agilent). Paired-end, strand-specific RNA-seq library was prepared using KAPA RNA Hyper Prep kit (KAPA Biosystems, Wilmington, MA), following the instructions (50). Resulting libraries were analyzed on an Agilent Bioanalyzer DNA 1000 chip (Agilent). Sequencing was performed on a Hiseq 2500 sequencer at the Genomics Core facility of University of California, San Diego, CA.

### RNA-Seq data processing

The RNA-seq pipeline used to analyze and perform QC/QA has been described in detail previously (51). Briefly, the sequences were aligned to the reference genomes using Bowtie2 (52). The aligned sequences were assigned to ORFs using HTSeq-counts (53). Differential expression analysis was performed using DESeq2 with a P-value threshold of 0.05 and an absolute fold-change threshold of 2 (54). To create the final counts matrix, counts from conserved genes in LAC samples were represented by the corresponding ortholog in TCH1516. The counts for accessory genes were filled with 0s if the genes were not present in the strain (i.e., LAC-specific genes had counts of 0 in TCH1516 samples and vice versa). Finally, to reduce the effect of noise, genes with average counts per sample <10 were removed. The final counts matrix with 2,581 genes was used to calculate transcripts per million (TPM).

### Metabolic modeling and assessment of significant differences in flux distribution

We used BiGG model iYS854 and set the lower bound to the corresponding nutrient exchange to −1 mmol/gDW/h (the negative sign is a modeling convention to allow for the influx of nutrients) and −13 mmol/gDW/h for oxygen exchange (as measured experimentally) (33). Next, we compared two conditions with (i) amino acid-rich medium and (ii) amino acid-poor medium. In the first condition, assuming that in the presence of amino acid, CodY mediates the repression of multiple target genes. Specifically, we turned off various combinations of amino acid transporters as reported in Table S4 by setting both the minimum and maximum bounds through their corresponding exchange reactions to 0. No regulatory constraints were added. Flux-balance analysis (FBA) was implemented with the biomass formation set as the functional network objective. Next, flux balance analysis was run in both conditions and the fluxes were sampled 10,000 times. All reaction fluxes were normalized by dividing by the growth rate to account for growth differences across the two media types. The flux distribution for each metabolic process was compared across both conditions using the Kolmogorov-Smirnov test, a non-parametric test which compares two continuous probability distributions. The distribution across two reactions was deemed to be significantly different when the Kolmogorov-Smirnoff statistic was larger than 0.99 with an adjusted *P*-value < 0.01.

### Motif mutation analysis

To gain insights into the CodY motifs from TCH1516 and LAC strains, MAGGIE was used for motif mutation analysis (23). For CodY, given a pair of CodY-binding sequences of the same target gene from TCH1516 and LAC strains, the peak sequence with a higher binding intensity was considered as a positive sequence; the other one with a lower binding intensity was considered as a negative sequence. Here, CodY has 135 pairs of positive and negative sequences from TCH1516 and LAC. MAGGIE computes differences of representative motif scores (i.e., motif mutations) within each sequence pair by subtracting the maximal scores of negative from positive sequences and then statistically tests for the association between motif mutations and the differences in CodY-binding intensity. Positive significant p-values from MAGGIE indicate that higher-affinity motif is associated with stronger CodY binding.

## Data Availability

The ChIP-exo and RNA-seq data sets are accessible through GEO under accession numbers GSE159856 and GSE163312.

## SUPPLEMENTAL MATERIAL

Fig S1 msystems.00279-23-s0001.eps Comparison of CodY sequences among *S. aureus* USA300, *L. monocytogenes*, and *B. subtilis*. Red rectangle denotes the CodY DNA-binding domain (Helix-Turn-Helix domain).

Fig S2 msystems.00279-23-s0002.eps Comparison of CodY sequences between *S. aureus* USA300 TCH1516 and LAC strains. The sequence alignment shows identical CodY amino acid residues in two distinct but related *S. aureus* strains.

Fig S3 msystems.00279-23-s0003.eps Comparison of CodY-binding intensity between TCH1516 and LAC strains. Though the genome-wide CodY-binding sites identified were the same in the two strains, strain-specific binding peaks had differing intensities.

Fig S4 msystems.00279-23-s0004.eps *S. aureus* USA300 CodY-binding motif from a merged set of TCH1516 and LAC binding peaks. There is a 20-bp CodY motif based on a merged set of CodY-binding peaks from the strains using MEME (E-value = 1.5e-082).

Fig S5 msystems.00279-23-s0005.eps Distribution of CodY peaks based on the range of motif score differences. X-axis represents the range of motif score differences. Y-axis represents the number of peaks that fall into each range. Black-color bar shows 45 peaks with the motif score difference at the range of (-1, 1).

Fig S6 msystems.00279-23-s0006.eps Comparison of genes in the CodY regulon (red) and CodY iModulon (blue). The number in the circle represents the amount of genes in the category. CodY regulon is defined by a binding peak in the genes' promoter region and detection of differential gene expression of the wild type and the *codY* mutant during growth in RPMI 1640 and 10% LB medium.

Table S1 msystems.00279-23-s0007.docx The strains used in this study.

Table S2 msystems.00279-23-s0008.docx The primers and plasmids used in this study.

Table S3 msystems.00279-23-s0009.docx The binding sites of CodY in *S. aureus* USA300 LAC.

Table S4 msystems.00279-23-s0010.docx The combinations of amino acids and transporters in the model.

ASM does not own the copyrights to Supplemental Material that may be linked to, or accessed through, an article. The authors have granted ASM a non-exclusive, world-wide license to publish the Supplemental Material files. Please contact the corresponding author directly for reuse.
