# Supplementary material for: Elucidating the CodY regulon in Staphylococcus aureus USA300 substrains TCH1516 and LAC
Source: mSystems. 2023 Jun 13;8(4):e00279-23. doi: 10.1128/msystems.00279-23 (PMC10470025; doi:10.1128/msystems.00279-23)
Supplement: Table S1 — The strains used in this study. [file msystems.00279-23-s0007.docx]

| **Strain** | **Genotype/Characteristics** | **Source or reference** |
| --- | --- | --- |
| *Staphylococcus aureus* USA300 TCH1516 | Community-associated methicillin-resistant *Staphylococcus aureus* USA300 clinical strain isolated from an outbreak in Houston | This study |
| *Staphylococcus aureus* USA300 LAC | Community-associated methicillin-resistant *Staphylococcus aureus* USA300 clinical strain isolated from the Los Angeles County jail | This study |
| *Staphylococcus aureus* USA300 JE2 | It was used as a parental strain for Nebraska Transposon Mutant Library, and it was derived from USA300 LAC that cured of its native plasmid. | University of Nebraska Medical Center |
| *codY* mutant | Gene *codY* was disrupted by the insertion of the mariner Tn mutagenesis | University of Nebraska Medical Center |
| *Escherichia coli* BL21(DE3) | *E. coli* str. B F^–^ *ompT* *gal* *dcm* *lon* *hsdS_B_*(*r_B_*^–^*m_B_*^–^) λ(DE3) | This study |
