# Supplementary material for: Elucidating the CodY regulon in Staphylococcus aureus USA300 substrains TCH1516 and LAC
Source: mSystems. 2023 Jun 13;8(4):e00279-23. doi: 10.1128/msystems.00279-23 (PMC10470025; doi:10.1128/msystems.00279-23)
Supplement: Table S2 — The primers and plasmids used in this study. [file msystems.00279-23-s0008.docx]

| **Oligonucleotides** | **Description/Sequence (5’-3’)** | **Source or reference** |
| --- | --- | --- |
| Plasmid |  |  |
| pET-30a(+) | Expression vector with *S. aureus* *codY* His tagged protein | EMD Millipore |
| Primers |  |  |
| T7_forward_primer | TAATACGACTCACTATAGGG | This study |
| T7_reverse_primer | TGCTAGTTATTGCTCAGCGG | This study |
| Adaptors |  |  |
| First adaptor sense oligo | Phosphate-GTGACTGGAGTTCAGACGTGCTCTTCCGATCT | This study |
| First adaptor antisense oligo | GATCGGAAGAGCACACGTCTGAACTCCAGTCACTT | This study |
| Gene |  |  |
| *codY* coding sequence | ATGAGCTTATTATCTAAAACGAGAGAGTTAAACACGTTACTTCAAAAACACAAAGGTATTGCGGTTGATTTTAAAGATGTAGCACAAACGATTAGTAGCGTAACTGTAACAAATGTATTTATTGTATCGCGTCGAGGTAAAATTTTAGGATCGAGTCTAAATGAATTATTAAAAAGTCAAAGAATTATTCAAATGTTGGAAGAAAGACATATTCCAAGTGAATATACAGAACGATTAATGGAAGTTAAACAAACAGAATCAAATATTGATATCGACAATGTATTAACAGTATTCCCACCTGAAAACAGAGAATTATTCATAGATAGTCGTACAACTATCTTCCCAATTTTAGGTGGAGGGGAAAGATTAGGTACATTAGTACTTGGTCGAGTACATGATGATTTTAATGAAAATGATTTGGTACTAGGTGAATATGCTGCTACAGTTATTGGTATGGAAATCTTACGTGAGAAGCATAGTGAAGTAGAAAAAGAAGCGCGCGATAAAGCTGCTATTACAATGGCAATTAATTCATTATCTTATTCTGAAAAAGAAGCGATTGAACATATCTTTGAAGAACTTGGCGGTACGGAAGGCCTATTAATCGCATCAAAAGTTGCAGATAGAGTTGGTATTACTAGATCTGTAATTGTAAATGCACTACGTAAATTAGAAAGTGCTGGTGTAATTGAATCACGTTCTTTAGGAATGAAAGGTACTTTCATTAAAGTTAAAAAAGAAAAATTCTTAGATGAATTAGAAAAAAGTAAATAA | This study |
| Upstream primer | CTCGATTCTATTAACAAGGG | This study |
| Buster primer | GCTTTTTCTAAATGTTTTTTAAGTAAATCAAGTAC | This study |
| Left CodY confirmation primer | CACAAAGGTATTGCGGTTGA | This study |
| Right CodY confirmation primer | CGTGATTCAATTACACCAGCA | This study |
