# Supplementary material for: Elucidating the CodY regulon in Staphylococcus aureus USA300 substrains TCH1516 and LAC
Source: mSystems. 2023 Jun 13;8(4):e00279-23. doi: 10.1128/msystems.00279-23 (PMC10470025; doi:10.1128/msystems.00279-23)
Supplement: Table S3 — The binding sites of CodY in S. aureus USA300 LAC. [file msystems.00279-23-s0009.docx]

| **No** | **Start** | **End** | **Locus tag (LAC)** | | **Locus tag (TCH1516)** | **Gene** | **Location** | **Intensity** |
| --- | --- | --- | --- | --- | --- | --- | --- | --- |
| 1 | 25225 | 25265 | ERW10_00160 | USA300HOU_RS10525 | | *eap* | regulatory | 12.3 |
| 2 | 45265 | 45306 | ERW10_00280 | USA300HOU_RS10410 | | N/A | regulatory | 3.2 |
| 3 | 64797 | 64832 | ERW10_00360 | USA300HOU_RS10330 | | *putP* | regulatory | 2.1 |
| 4 | 64797 | 64832 | ERW10_00365 | USA300HOU_RS10325 | | *gatC* | regulatory | 2.1 |
| 5 | 166375 | 166415 | ERW10_01030 | USA300HOU_RS09650 | | *splB* | regulatory | 12.3 |
| 6 | 253587 | 253620 | ERW10_01450 | USA300HOU_RS09225 | | N/A | regulatory | 13.6 |
| 7 | 258320 | 258350 | ERW10_01470 | USA300HOU_RS09205 | | N/A | regulatory | 11.8 |
| 8 | 258320 | 258350 | ERW10_01475 | USA300HOU_RS09200 | | *acsA* | regulatory | 11.8 |
| 9 | 271684 | 271715 | ERW10_01520 | USA300HOU_RS09155 | | N/A | regulatory | 15.6 |
| 10 | 275848 | 275880 | ERW10_01535 | USA300HOU_RS09140 | | N/A | regulatory | 19.6 |
| 11 | 287875 | 287910 | ERW10_01600 | USA300HOU_RS09070 | | N/A | regulatory | 8.7 |
| 12 | 287875 | 287910 | ERW10_01605 | USA300HOU_RS09065 | | *ald* | regulatory | 8.7 |
| 13 | 393014 | 393056 | ERW10_02090 | USA300HOU_RS08570 | | *pxpB* | intragenic | 8.5 |
| 14 | 562783 | 562815 | ERW10_03100 | USA300HOU_RS07550 | | *rpsA* | regulatory | 3.6 |
| 15 | 571002 | 571035 | ERW10_03145 | USA300HOU_RS07500 | | *aroC* | regulatory | 12.5 |
| 16 | 578031 | 578072 | ERW10_03180 | USA300HOU_RS07465 | | N/A | regulatory | 2.4 |
| 17 | 613198 | 613230 | ERW10_03305 | USA300HOU_RS07340 | | *ebh* | intragenic | 12.9 |
| 18 | 657480 | 657527 | ERW10_03420 | USA300HOU_RS07210 | | N/A | intragenic | 2.4 |
| 19 | 664648 | 664678 | ERW10_03450 | USA300HOU_RS07180 | | *brnQ* | regulatory | 2.6 |
| 20 | 678005 | 678035 | ERW10_03520 | USA300HOU_RS07100 | | N/A | regulatory | 9.8 |
| 21 | 679677 | 679717 | ERW10_03525 | USA300HOU_RS07095 | | N/A | regulatory | 9.5 |
| 22 | 702572 | 702605 | ERW10_03640 | USA300HOU_RS06975 | | *trpC* | regulatory | 12.4 |
| 23 | 705700 | 705743 | ERW10_03655 | USA300HOU_RS06960 | | N/A | regulatory | 118.7 |
| 24 | 707248 | 707278 | ERW10_03660 | USA300HOU_RS06955 | | N/A | regulatory | 5.2 |
| 25 | 707248 | 707278 | ERW10_03665 | USA300HOU_RS06950 | | N/A | regulatory | 5.2 |
| 26 | 717423 | 717463 | ERW10_03705 | USA300HOU_RS06910 | | N/A | regulatory | 4.6 |
| 27 | 746050 | 746090 | ERW10_03820 | USA300HOU_RS06785 | | N/A | regulatory | 8.4 |
| 28 | 746050 | 746090 | ERW10_03825 | USA300HOU_RS06780 | | N/A | regulatory | 8.4 |
| 29 | 752626 | 752666 | ERW10_03855 | USA300HOU_RS06750 | | N/A | regulatory | 10.5 |
| 30 | 752626 | 752666 | ERW10_03860 | USA300HOU_RS06745 | | N/A | regulatory | 10.5 |
| 31 | 769192 | 769242 | ERW10_03985 | USA300HOU_RS06610 | | N/A | regulatory | 5.5 |
| 32 | 773794 | 773839 | ERW10_04010 | USA300HOU_RS06585 | | N/A | regulatory | 3.2 |
| 33 | 777777 | 777814 | ERW10_04035 | USA300HOU_RS06560 | | *miaA* | intragenic | 2.8 |
| 34 | 839574 | 839615 | ERW10_04295 | USA300HOU_RS06300 | | *xerC* | regulatory | 3.1 |
| 35 | 934596 | 934645 | ERW10_04725 | USA300HOU_RS05860 | | *argF* | regulatory | 9.2 |
| 36 | 934596 | 934645 | ERW10_04730 | USA300HOU_RS05855 | | N/A | regulatory | 9.2 |
| 37 | 987173 | 987213 | ERW10_05000 | USA300HOU_RS05560 | | N/A | regulatory | 10.6 |
| 38 | 1003801 | 1003837 | ERW10_05090 | USA300HOU_RS05470 | | *lpdA* | intragenic | 1.5 |
| 39 | 1033465 | 1033495 | ERW10_05230 | USA300HOU_RS05320 | | *purQ* | intragenic | 9.4 |
| 40 | 1039218 | 1039250 | ERW10_05270 | USA300HOU_RS05280 | | *qoxA* | regulatory | 4.8 |
| 41 | 1053758 | 1053790 | ERW10_05330 | USA300HOU_RS05220 | | N/A | regulatory | 6.8 |
| 42 | 1072919 | 1072950 | ERW10_05425 | USA300HOU_RS05125 | | N/A | regulatory | 13.6 |
| 43 | 1076263 | 1076295 | ERW10_05460 | USA300HOU_RS05095 | | N/A | regulatory | 11.5 |
| 44 | 1076263 | 1076295 | ERW10_05465 | USA300HOU_RS05090 | | N/A | regulatory | 11.5 |
| 45 | 1093531 | 1093571 | ERW10_05535 | USA300HOU_RS05020 | | N/A | regulatory | 13.8 |
| 46 | 1093531 | 1093571 | ERW10_05540 | USA300HOU_RS05015 | | N/A | regulatory | 13.8 |
| 47 | 1109191 | 1109231 | ERW10_05610 | USA300HOU_RS04945 | | *spxA* | regulatory | 9.6 |
| 48 | 1109191 | 1109231 | ERW10_05615 | USA300HOU_RS04940 | | *trpS* | regulatory | 9.6 |
| 49 | 1116078 | 1116118 | ERW10_05640 | USA300HOU_RS04915 | | N/A | regulatory | 13.8 |
| 50 | 1121945 | 1121985 | ERW10_05665 | USA300HOU_RS04890 | | N/A | regulatory | 12.5 |
| 51 | 1121945 | 1121985 | ERW10_05670 | USA300HOU_RS04885 | | N/A | regulatory | 12.5 |
| 52 | 1127870 | 1127907 | ERW10_05700 | USA300HOU_RS04855 | | N/A | intragenic | 5.8 |
| 53 | 1129342 | 1129382 | ERW10_05710 | USA300HOU_RS04845 | | N/A | intragenic | 6.8 |
| 54 | 1151322 | 1151362 | ERW10_05780 | USA300HOU_RS04775 | | N/A | regulatory | 11.5 |
| 55 | 1151322 | 1151362 | ERW10_05785 | USA300HOU_RS04770 | | N/A | regulatory | 11.5 |
| 56 | 1203268 | 1203303 | ERW10_06065 | USA300HOU_RS04490 | | N/A | intragenic | 3.2 |
| 57 | 1211955 | 1211990 | ERW10_06135 | USA300HOU_RS04425 | | *sek* | regulatory | 3.5 |
| 58 | 1215619 | 1215660 | ERW10_06150 | USA300HOU_RS04410 | | N/A | regulatory | 8.3 |
| 59 | 1216979 | 1217021 | ERW10_06155 | USA300HOU_RS04405 | | N/A | regulatory | 7.2 |
| 60 | 1223810 | 1223850 | ERW10_06210 | USA300HOU_RS04340 | | N/A | regulatory | 11.2 |
| 61 | 1253862 | 1253892 | ERW10_06375 | USA300HOU_RS04180 | | N/A | regulatory | 3.7 |
| 62 | 1279555 | 1279585 | ERW10_06480 | USA300HOU_RS04065 | | *raiA* | regulatory | 7.3 |
| 63 | 1280717 | 1280747 | ERW10_06490 | USA300HOU_RS04055 | | N/A | intragenic | 8.5 |
| 64 | 1304624 | 1304656 | ERW10_06610 | USA300HOU_RS03930 | | N/A | regulatory | 10.3 |
| 65 | 1310094 | 1310134 | ERW10_06630 | USA300HOU_RS03910 | | *hisC* | regulatory | 12.6 |
| 66 | 1346425 | 1346465 | ERW10_06800 | USA300HOU_RS03730 | | N/A | regulatory | 3.8 |
| 67 | 1362962 | 1362992 | ERW10_06905 | USA300HOU_RS03620 | | N/A | intragenic | 6.3 |
| 68 | 1389108 | 1389148 | ERW10_07025 | USA300HOU_RS03495 | | N/A | regulatory | 15.8 |
| 69 | 1445348 | 1445378 | ERW10_07325 | USA300HOU_RS03165 | | N/A | regulatory | 3.2 |
| 70 | 1499803 | 1499833 | ERW10_07580 | USA300HOU_RS02910 | | N/A | regulatory | 8.5 |
| 71 | 1614012 | 1614050 | ERW10_08160 | USA300HOU_RS02375 | | *gltB* | regulatory | 10.5 |
| 72 | 1614012 | 1614050 | ERW10_08165 | USA300HOU_RS02370 | | N/A | regulatory | 10.5 |
| 73 | 1622873 | 1622910 | ERW10_08210 | USA300HOU_RS02325 | | N/A | regulatory | 5.0 |
| 74 | 1640024 | 1640059 | ERW10_08310 | USA300HOU_RS02240 | | *lpl10* | regulatory | 3.3 |
| 75 | 1645012 | 1645050 | ERW10_08340 | USA300HOU_RS02210 | | *lpl4* | intragenic | 4.3 |
| 76 | 1647960 | 1647998 | ERW10_08355 | USA300HOU_RS02195 | | *lpl1* | regulatory | 12.6 |
| 77 | 1647960 | 1647998 | ERW10_08360 | USA300HOU_RS02190 | | *spn* | regulatory | 12.6 |
| 78 | 1667871 | 1667901 | ERW10_08465 | USA300HOU_RS02080 | | N/A | regulatory | 5.8 |
| 79 | 1686450 | 1686495 | ERW10_08560 | USA300HOU_RS01985 | | N/A | intragenic | 7.8 |
| 80 | 1696398 | 1696440 | ERW10_08630 | USA300HOU_RS01910 | | N/A | regulatory | 11.3 |
| 81 | 1697849 | 1697885 | ERW10_08635 | USA300HOU_RS01905 | | N/A | intragenic | 26.6 |
| 82 | 1714033 | 1714073 | ERW10_08710 | USA300HOU_RS01825 | | *efeB* | intragenic | 2.5 |
| 83 | 1716141 | 1716178 | ERW10_08720 | USA300HOU_RS01815 | | N/A | regulatory | 9.3 |
| 84 | 1726256 | 1726288 | ERW10_08770 | USA300HOU_RS01765 | | N/A | intragenic | 8.4 |
| 85 | 1739339 | 1739379 | ERW10_08835 | USA300HOU_RS01700 | | *lip2* | regulatory | 9.6 |
| 86 | 1739339 | 1739379 | ERW10_08840 | USA300HOU_RS01695 | | N/A | regulatory | 9.6 |
| 87 | 1741811 | 1741841 | ERW10_08850 | USA300HOU_RS01685 | | N/A | intragenic | 2.5 |
| 88 | 1754425 | 1754465 | ERW10_08900 | USA300HOU_RS01630 | | N/A | regulatory | 9.6 |
| 89 | 1754425 | 1754465 | ERW10_08905 | USA300HOU_RS01625 | | *brnQ* | regulatory | 9.6 |
| 90 | 1764331 | 1764371 | ERW10_08975 | USA300HOU_RS01560 | | N/A | intragenic | 4.6 |
| 91 | 1807998 | 1808038 | ERW10_09190 | USA300HOU_RS01340 | | *tarS* | regulatory | 5.7 |
| 92 | 1816930 | 1816970 | ERW10_09225 | USA300HOU_RS01305 | | N/A | regulatory | 18.0 |
| 93 | 1823020 | 1823060 | ERW10_09260 | USA300HOU_RS01270 | | N/A | intragenic | 9.8 |
| 94 | 1828971 | 1829021 | ERW10_09280 | USA300HOU_RS01245 | | N/A | regulatory | 9.7 |
| 95 | 1828971 | 1829021 | ERW10_09290 | USA300HOU_RS01235 | | N/A | regulatory | 9.7 |
| 96 | 1834306 | 1834341 | ERW10_09310 | USA300HOU_RS01215 | | N/A | regulatory | 7.8 |
| 97 | 1834306 | 1834341 | ERW10_09315 | USA300HOU_RS01210 | | N/A | regulatory | 7.8 |
| 98 | 1874534 | 1874575 | ERW10_09460 | USA300HOU_RS01055 | | N/A | regulatory | 11.5 |
| 99 | 1874534 | 1874575 | ERW10_09465 | USA300HOU_RS01050 | | N/A | regulatory | 11.5 |
| 100 | 1876491 | 1876531 | ERW10_09470 | USA300HOU_RS01045 | | N/A | intragenic | 16.5 |
| 101 | 1877968 | 1878003 | ERW10_09475 | USA300HOU_RS01040 | | N/A | intragenic | 14.5 |
| 102 | 1892673 | 1892703 | ERW10_09535 | USA300HOU_RS00980 | | *brnQ* | regulatory | 8.6 |
| 103 | 1915942 | 1915980 | ERW10_09610 | USA300HOU_RS00905 | | N/A | regulatory | 1.9 |
| 104 | 1915942 | 1915980 | ERW10_09615 | USA300HOU_RS00900 | | N/A | regulatory | 1.9 |
| 105 | 1940182 | 1940217 | ERW10_09720 | USA300HOU_RS00795 | | *adhE* | regulatory | 9.0 |
| 106 | 1957244 | 1957282 | ERW10_09810 | USA300HOU_RS00700 | | N/A | regulatory | 4.0 |
| 107 | 1964247 | 1964278 | ERW10_09845 | USA300HOU_RS00665 | | N/A | regulatory | 70.1 |
| 108 | 1985164 | 1985199 | ERW10_09930 | USA300HOU_RS00575 | | N/A | regulatory | 3.5 |
| 109 | 2006889 | 2006929 | ERW10_10010 | USA300HOU_RS00500 | | N/A | intragenic | 10.2 |
| 110 | 2010988 | 2011022 | ERW10_10030 | USA300HOU_RS00480 | | N/A | regulatory | 3.1 |
| 111 | 2010988 | 2011022 | ERW10_10040 | USA300HOU_RS00470 | | N/A | regulatory | 3.1 |
| 112 | 2048178 | 2048218 | ERW10_10230 | USA300HOU_RS00275 | | N/A | regulatory | 16.5 |
| 113 | 2048178 | 2048218 | ERW10_10235 | USA300HOU_RS00270 | | N/A | regulatory | 16.5 |
| 114 | 2096248 | 2096288 | ERW10_10455 | USA300HOU_RS00060 | | N/A | regulatory | 12.6 |
| 115 | 2138147 | 2138187 | ERW10_10665 | USA300HOU_RS14585 | | N/A | regulatory | 12.4 |
| 116 | 2138147 | 2138187 | ERW10_10670 | USA300HOU_RS14580 | | N/A | regulatory | 12.4 |
| 117 | 2144196 | 2144235 | ERW10_10700 | USA300HOU_RS14550 | | N/A | regulatory | 37.8 |
| 118 | 2156443 | 2156483 | ERW10_10760 | USA300HOU_RS14490 | | *icaB* | intragenic | 9.4 |
| 119 | 2199440 | 2199480 | ERW10_10915 | USA300HOU_RS14335 | | *aur* | regulatory | 2.4 |
| 120 | 2201068 | 2201099 | ERW10_10920 | USA300HOU_RS14330 | | N/A | intragenic | 5.2 |
| 121 | 2207770 | 2207800 | ERW10_10955 | USA300HOU_RS14295 | | *clfB* | intragenic | 3.5 |
| 122 | 2219122 | 2219166 | ERW10_11010 | USA300HOU_RS14235 | | N/A | regulatory | 44.3 |
| 123 | 2220028 | 2220066 | ERW10_11015 | USA300HOU_RS14230 | | N/A | regulatory | 6.0 |
| 124 | 2236884 | 2236914 | ERW10_11105 | USA300HOU_RS14140 | | N/A | regulatory | 8.3 |
| 125 | 2242765 | 2242801 | ERW10_11130 | USA300HOU_RS14115 | | N/A | regulatory | 7.9 |
| 126 | 2245331 | 2245370 | ERW10_11135 | USA300HOU_RS14110 | | N/A | regulatory | 8.6 |
| 127 | 2245331 | 2245370 | ERW10_11140 | USA300HOU_RS14100 | | N/A | regulatory | 8.6 |
| 128 | 2270191 | 2270230 | ERW10_11295 | USA300HOU_RS13945 | | N/A | regulatory | 19.5 |
| 129 | 2282959 | 2282999 | ERW10_11345 | USA300HOU_RS13895 | | N/A | regulatory | 18.9 |
| 130 | 2299774 | 2299814 | ERW10_11410 | USA300HOU_RS13830 | | *clpL* | regulatory | 7.4 |
| 131 | 2299774 | 2299814 | ERW10_11415 | USA300HOU_RS13825 | | N/A | regulatory | 7.4 |
| 132 | 2303997 | 2304037 | ERW10_11430 | USA300HOU_RS13810 | | N/A | regulatory | 25.4 |
| 133 | 2323143 | 2323185 | ERW10_11545 | USA300HOU_RS13695 | | N/A | regulatory | 26.8 |
| 134 | 2323143 | 2323185 | ERW10_11550 | USA300HOU_RS13690 | | N/A | regulatory | 26.8 |
| 135 | 2335500 | 2335540 | ERW10_11605 | USA300HOU_RS13630 | | N/A | regulatory | 22.6 |
| 136 | 2381962 | 2381999 | ERW10_11820 | USA300HOU_RS13405 | | N/A | regulatory | 8.9 |
| 137 | 2397798 | 2397833 | ERW10_11910 | USA300HOU_RS13315 | | N/A | intragenic | 3.6 |
| 138 | 2429664 | 2429699 | ERW10_12050 | USA300HOU_RS13155 | | N/A | regulatory | 3.6 |
| 139 | 2444643 | 2444683 | ERW10_12110 | USA300HOU_RS13085 | | *sbi* | regulatory | 9.6 |
| 140 | 2457643 | 2457681 | ERW10_12205 | USA300HOU_RS12990 | | N/A | regulatory | 2.3 |
| 141 | 2477269 | 2477306 | ERW10_12285 | USA300HOU_RS12910 | | N/A | intragenic | 10.0 |
| 142 | 2494442 | 2494482 | ERW10_12375 | USA300HOU_RS12815 | | N/A | regulatory | 5.6 |
| 143 | 2497276 | 2497299 | ERW10_12385 | USA300HOU_RS12805 | | N/A | regulatory | 5.1 |
| 144 | 2504292 | 2504322 | ERW10_12415 | USA300HOU_RS12775 | | N/A | intragenic | 3.6 |
| 145 | 2517572 | 2517610 | ERW10_12480 | USA300HOU_RS12705 | | N/A | regulatory | 8.7 |
| 146 | 2536645 | 2536685 | ERW10_12580 | USA300HOU_RS12605 | | N/A | regulatory | 12.6 |
| 147 | 2545637 | 2545677 | ERW10_12615 | USA300HOU_RS12570 | | N/A | regulatory | 22.6 |
| 148 | 2545637 | 2545677 | ERW10_12620 | USA300HOU_RS12565 | | N/A | regulatory | 22.6 |
| 149 | 2548358 | 2548398 | ERW10_12645 | USA300HOU_RS12540 | | N/A | regulatory | 3.5 |
| 150 | 2569800 | 2569840 | ERW10_12740 | USA300HOU_RS12440 | | N/A | regulatory | 11.7 |
| 151 | 2569800 | 2569840 | ERW10_12745 | USA300HOU_RS12435 | | N/A | regulatory | 11.7 |
| 152 | 2573654 | 2573694 | ERW10_12760 | USA300HOU_RS12415 | | *sarR* | regulatory | 12.6 |
| 153 | 2580663 | 2580707 | ERW10_12805 | USA300HOU_RS12370 | | N/A | regulatory | 6.5 |
| 154 | 2644923 | 2644963 | ERW10_13220 | USA300HOU_RS11940 | | *hysA* | intragenic | 15.6 |
| 155 | 2653646 | 2653679 | ERW10_13275 | USA300HOU_RS11885 | | *lacC* | intragenic | 10.9 |
| 156 | 2697744 | 2697780 | ERW10_13500 | USA300HOU_RS11655 | | N/A | intragenic | 5.5 |
| 157 | 2714579 | 2714613 | ERW10_13535 | USA300HOU_RS11620 | | N/A | regulatory | 30.8 |
| 158 | 2733078 | 2733108 | ERW10_13630 | USA300HOU_RS11520 | | N/A | regulatory | 7.3 |
| 159 | 2733078 | 2733108 | ERW10_13635 | USA300HOU_RS11515 | | N/A | regulatory | 7.3 |
| 160 | 2743451 | 2743491 | ERW10_13680 | USA300HOU_RS11465 | | N/A | regulatory | 3.7 |
| 161 | 2794913 | 2794957 | ERW10_13950 | USA300HOU_RS11180 | | *alr* | intragenic | 1.5 |
| 162 | 2815662 | 2815703 | ERW10_14045 | USA300HOU_RS11075 | | *ilvC* | regulatory | 7.5 |
| 163 | 2819737 | 2819778 | ERW10_14060 | USA300HOU_RS11060 | | *ilvD* | regulatory | 26.9 |
| 164 | 2819737 | 2819778 | ERW10_14070 | USA300HOU_RS11050 | | *tsaE* | regulatory | 26.9 |
| 165 | 2829305 | 2829343 | ERW10_14120 | USA300HOU_RS11000 | | N/A | regulatory | 39.9 |

N/A denotes the gene name unavailable in the reference genome.
