# Supplementary material for: Elucidating the CodY regulon in Staphylococcus aureus USA300 substrains TCH1516 and LAC
Source: mSystems. 2023 Jun 13;8(4):e00279-23. doi: 10.1128/msystems.00279-23 (PMC10470025; doi:10.1128/msystems.00279-23)
Supplement: Table S4 — The combinations of amino acids and transporters in the model. [file msystems.00279-23-s0010.docx]

**Table S4 The combinations of amino acids and transporters in the model**

| **Amino Acids** | **Transporters** |
| --- | --- |
| isoleucine | ILEt2r |
| leucine | LEUt2r |
| valine | VALt2r |
| aspartate | ASPabc, ASPt2, ASPt3, ASPT4 |
| asparagine | ASNt2r |
